# A Review on Deep Learning Methods for Glioma Segmentation, Limitations, and Future Perspectives

**DOI:** 10.3390/jimaging11080269

**Published:** 2025-08-11

**Authors:** Cecilia Diana-Albelda, Álvaro García-Martín, Jesus Bescos

**Affiliations:** Video Processing and Understanding Lab, Escuela Politécnica Superior, Universidad Autónoma de Madrid, 28049 Madrid, Spain; alvaro.garcia@uam.es (Á.G.-M.); j.bescos@uam.es (J.B.)

**Keywords:** brain tumor, deep learning, glioma, hardware resources, practical medical deployment, semantic segmentation

## Abstract

Accurate and automated segmentation of gliomas from Magnetic Resonance Imaging (MRI) is crucial for effective diagnosis, treatment planning, and patient monitoring. However, the aggressive nature and morphological complexity of these tumors pose significant challenges that call for advanced segmentation techniques. This review provides a comprehensive analysis of Deep Learning (DL) methods for glioma segmentation, with a specific focus on bridging the gap between research performance and practical clinical deployment. We evaluate over 80 state-of-the-art models published up to 2025, categorizing them into CNN-based, Pure Transformer, and Hybrid CNN-Transformer architectures. The primary objective of this paper is to critically assess these models not only on their segmentation accuracy but also on their computational efficiency and suitability for real-world medical environments by incorporating hardware resource considerations. We present a comparison of model performance on the BraTS datasets benchmark and introduce a suitability analysis for top-performing models based on their robustness, efficiency, and completeness of tumor region delineation. By identifying current trends, limitations, and key trade-offs, this review offers future research directions aimed at optimizing the balance between technical performance and clinical usability to improve diagnostic outcomes for glioma patients.

## 1. Introduction

Gliomas are among the most aggressive and lethal primary brain tumors, characterized by low survival rates and irregular growth patterns [1]. Accurate segmentation of these tumors is essential for diagnosis, treatment planning, and monitoring disease progression [2,3]. However, manual segmentation performed by radiologists is time-consuming, labor-intensive, and prone to inter-observer variability [4]. Given the importance of early and precise detection, the development of automated segmentation methods for this task has become a crucial research area [5,6].

Deep Learning (DL) techniques have emerged as a powerful tool for medical image analysis, including the semantic segmentation of brain tumors such as gliomas [7,8,9]. Current approaches in this field can be broadly categorized into three main types: (1) CNN-based methods, which excel in extracting spatial features; (2) Pure Transformer methods, which capture global context but require substantial computational resources; and (3) Hybrid CNN-Transformer, which aim to combine the strengths of both architectures.

Despite the significant progress achieved by these models, further research is needed to refine them, ensuring a balance between performance, computational efficiency, and clinical applicability, ultimately assisting radiologists in making faster and more reliable diagnoses.

Numerous surveys have explored brain tumor image analysis [10,11], distinguishing between classification [12,13,14] and segmentation tasks [15,16,17]. However, few have specifically focused on glioma segmentation [5,8,18] and these predominantly discuss Machine Learning and Deep Learning algorithms in a general context [6,19]. More importantly, the vast majority of the existing surveys share a fundamental limitation: they focus almost exclusively on segmentation accuracy while overlooking crucial details for real-world deployment, such as the number of trainable parameters and hardware requirements [20]. This omission creates a significant gap between state-of-the-art academic performance and the solutions that are genuinely feasible in a clinical environment.

To bridge this gap, the primary objective of this study is to provide a comprehensive and critical overview of the state-of-the-art, evaluating Deep Learning methods not only on their performance but also on their practical viability. To achieve this, our contributions are as follows:

- A comparison of more than 80 Deep Learning methods for glioma segmentation up to 2025, providing insights into their effectiveness and efficiency.
- An analysis of the number of tunable parameters of each method and their adaptability in clinical applications.
- An identification of current trends and limitations within the field, along with a proposal for future research directions and suggestions for improvement.

This review represents a step forward, as it fills a significant gap in the current literature by incorporating hardware considerations into the evaluation of Deep Learning methods for glioma segmentation. By doing so, it guides the development of feasible technologies that can realistically improve glioma diagnosis and treatment outcomes.

### Literature Search Strategy

To conduct this review, a structured and methodical approach has been adopted to identify, select, and analyze the most relevant scientific literature on glioma segmentation using deep learning techniques. The search focused on studies mainly published between 2020 and early 2025, with the aim of comparing the performance and efficiency of CNN-based, Transformer-based, and hybrid CNN-Transformer architectures.

We searched several major scientific databases, including IEEE Xplore, ScienceDirect, SpringerLink, and Wiley Online Library. These were complemented by queries in Google Scholar to capture recent conference proceedings, preprints, and emerging methods. The search strategy employed combinations of the following keywords:

- “glioma segmentation”;
- “brain tumor” AND “deep learning”;
- “glioma” AND “CNN”, “U-Net”, “Transformer”, “Vision Transformer”, “SAM”, “multimodal segmentation”, “survey”.

The selection process comprised two screening phases, resulting in the inclusion of over 80 peer-reviewed papers that form the foundation of our comparative analysis. First, we conducted a preliminary screening of titles and abstracts to exclude studies unrelated to glioma segmentation or those lacking a methodological contribution in deep learning. Second, a full-text evaluation was performed to retain studies that (a) focus specifically on glioma or brain tumor segmentation, (b) report experimental results on public benchmarks such as BraTS, and (c) provide quantitative performance metrics and model complexity indicators such as parameter count.

To ensure transparency and methodological rigor, our review approach was guided by the principles of the Preferred Reporting Items for Systematic Reviews and Meta-Analyses (PRISMA) framework [21], a widely recognized guideline designed to enhance the clarity, reproducibility, and comprehensiveness of literature reviews.

## 2. Glioma Segmentation

Glioma segmentation refers to the process of delineating tumor regions from brain Magnetic Resonance Imaging (MRI) scans, a crucial step in the diagnosis and treatment of gliomas [22].

**Figure 1:**
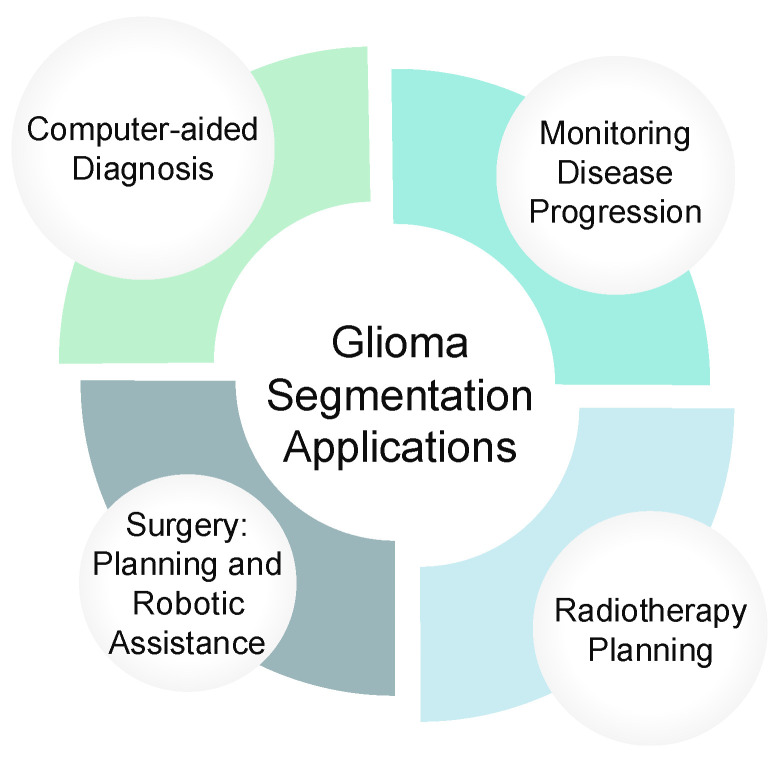
Clinical Applications of Glioma Segmentation. This diagram shows the medical applications of glioma segmentation, highlighting its role in: Computer-aided Diagnosis, Monitoring Disease Progression, Surgery (Planning and Robotic Assistance), and Radiotherapy Planning.

As illustrated in Figure 1, glioma segmentation has four main clinical applications: (1) Computer-aided Diagnosis, where AI-assisted tools help radiologists detect tumors; (2) Surgery Planning and Robotic Assistance, which rely on precise tumor boundaries to guide neurosurgical procedures; (3) Radiotherapy Planning, ensuring accurate targeting of radiation to tumor areas while sparing healthy tissue; and (4) Monitoring Disease Progression, enabling clinicians to track tumor evolution or response to treatment. Given the aggressive nature of many gliomas, robust segmentation methods are essential for improving patient outcomes [23].

### 2.1. Glioma

Gliomas are the most common primary tumors of the central nervous system (CNS), arising from glial or stem cells [24]. They are categorized into two types: infiltrating (diffuse) gliomas [25], which spread extensively into adjacent brain tissue, and circumscribed gliomas [26,27], which have clearer boundaries and are typically easier to treat.

Traditionally, the World Health Organization (WHO) classified gliomas from Grade I to IV based on their histological characteristics and degree of malignancy [28], with glioblastoma (GB) representing the most aggressive form (Grade IV), known for its rapid growth, necrosis, and resistance to standard therapies [29,30]. However, due to the limitations regarding inter-observer variability of purely histological classification, the 2021 WHO CNS tumor classification introduced a more robust, integrated diagnostic framework that combines histology with molecular and genetic markers [31].

The diverse characteristics of gliomas present significant challenges in treatment and image-based segmentation. Variability in tumor appearance, growth patterns, and different imaging techniques across institutions can hinder accurate diagnosis and treatment planning [32,33]. Deep Learning-driven automated segmentation tools are being developed to provide consistent and precise tumor delineation, aiding radiologists in decision-making and contributing to more efficient treatment.

### 2.2. MRI for Glioma Segmentation

MRI is the gold standard for glioma segmentation, providing detailed anatomical and functional insights into brain tumors [34]. Glioma imaging typically involves a multi-parametric MRI (mp-MRI) protocol that includes T1-weighted (T1), T2-weighted (T2), contrast-enhanced T1 (T1c), and Fluid-Attenuated Inversion Recovery T2 (T2-FLAIR) images, each highlighting distinct tissue properties [35]. In addition to these conventional sequences, some studies have also explored the potential contribution of diffusion-weighted imaging (DWI) [36], which provides information about water molecule motion and has shown promise when combined with anatomical and post-contrast images for segmentation tasks.

MRI images are acquired by exposing the patient to a strong magnetic field, followed by the application of radio-frequency pulses that interact with hydrogen protons (H+) in brain tissues [37]. The response of these protons generates the desired signal, with T1 and T2 images reflecting longitudinal and transverse relaxation times, respectively.

Accurate segmentation of gliomas requires integrating information from all conventional MRI modalities: T2 and T2-FLAIR help delineate peritumoral edema and infiltrative regions; T1c highlights active tumor areas, indicating regions of vascularization; and T1 assists in differentiating necrotic regions, a crucial factor in GB segmentation. Given these complementary sources of information, Deep Learning models for glioma segmentation must leverage multimodality integration, as relying on a single modality leads to suboptimal results [38].

## 3. Deep Learning Methods for Glioma Segmentation

Glioma segmentation methods based on Deep Learning can be categorized into three main groups: CNN-based, Pure Transformer, and Hybrid CNN-Transformer approaches.

CNN-based methods extract spatial features using convolutional layers, outperforming at local feature learning but struggling with long-range spatial dependencies. Pure Transformer methods, on the other hand, replace convolutions with self-attention, capturing global image context but demanding significantly more computational resources. Hybrid CNN-Transformer methods combine both approaches, aiming to balance precision and efficiency.

### 3.1. CNN-Based

**Figure 2:**
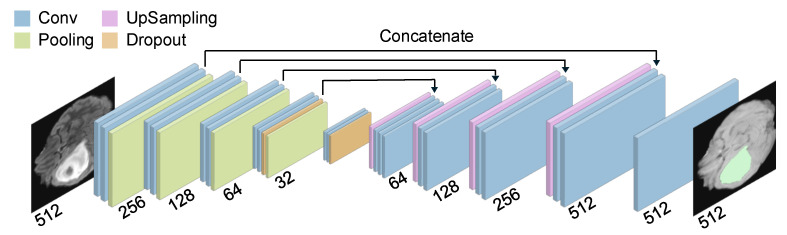
CNN Architecture. Utilizes convolution, pooling, and upsampling layers for feature extraction and segmentation.

Convolutional Neural Networks (CNNs), such as the U-Net architecture, are essential in medical image analysis, especially in glioma segmentation. As shown in Figure 2, these networks typically consist of convolutional layers for feature extraction, pooling to reduce spatial dimensions, and upsampling layers to restore the output images to the input size. Skip connections, which merge features from different layers, are a critical component for a detailed segmentation.

Among the CNN-based methods, 3D U-Net [39] and V-Net [40] focus on efficiently handling volumetric data, enhancing data management, and utilizing advanced loss functions to address class imbalances. Cascading CNNs, like those described in [41,42], and enhanced architectures like dResU-Net [43] and Dense Unet+ [44], incorporate techniques like residual blocks for robust feature extraction. However, they still exhibit a high dependence on the training dataset. In contrast, nnU-Net [45] dynamically adapts its architecture to each dataset, improving performance without requiring manual tuning. Additionally, methods like RFNet [46] and MAF-Net [47] offer adaptable solutions to the absence of specific modalities, while others employ innovative fusion techniques to better handle the multimodal nature of brain MRI data [48,49,50].

Recent innovations in glioma segmentation also include MM-BiFPN [51], which uses a fusion network with bidirectional pyramids, and GAM-Net [52], which integrates a dual convolution encoder and a gradient-driven decoder. Similarly, studies like [53,54] focus on leveraging variational auto-encoders and the Fuzzy Feature Contrast Maximization method to improve tumor delineation.

Despite these advances, the models still struggle with long-range dependencies, prompting the rise of Transformers in current segmentation techniques, as they effectively manage spatial and contextual relationships in medical images [47,55,56,57].

### 3.2. Pure Transformer

**Figure 3:**
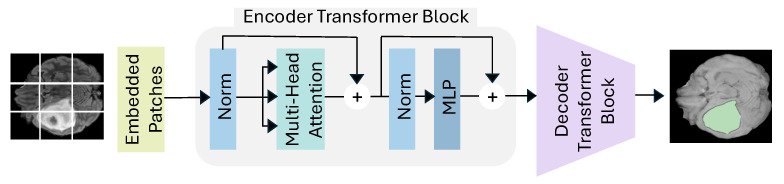
Pure Transformer Architecture. Incorporates multi-head attention blocks to capture long-range dependencies from volumetric image patches.

Figure 3 showcases the typical architecture of Pure Transformers for glioma segmentation. This approach processes images by converting them into sequences of patches and employs the Encoder Transformer Block, which includes multi-headed self-attention and position-wise feed-forward networks, to capture global dependencies across the entire brain scan. These Transformers utilize normalization layers and residual connections to enhance training stability, enabling a comprehensive understanding of the entire image, leveraging the strengths of attention mechanisms to produce highly accurate segmentation [58].

In the realm of Pure Transformer-based glioma segmentation, models like BTSwin U-Net [59] and Swin-Unet [60] enhance contextual information and spatial dependencies by utilizing Swin Transformers within U-shaped architectures. These models, while improving segmentation precision, require extensive training and substantial computational resources. Similarly, EMSViT [61] optimizes Transformer technology for medical imaging by integrating diverse convolution sizes for efficiency, while other alternatives [62,63,64] improve 3D data processing and resolution to preserve volumetric information.

Addressing more specialized challenges, MMCFormer [65] tackles the problem of missing modalities by employing co-training with 3D Transformer blocks to improve prediction reliability, even with incomplete datasets.

These models illustrate a trajectory towards optimizing Transformer technology for medical imaging, effectively capturing spatial and contextual information. However, they also incur a high computational burden, posing challenges for practical clinical application. To address this, the development of Hybrid CNN-Transformer architectures has emerged, aiming to balance the deep contextual insights of Transformers with the computational efficiency of CNNs.

### 3.3. Hybrid CNN-Transformer

**Figure 4:**
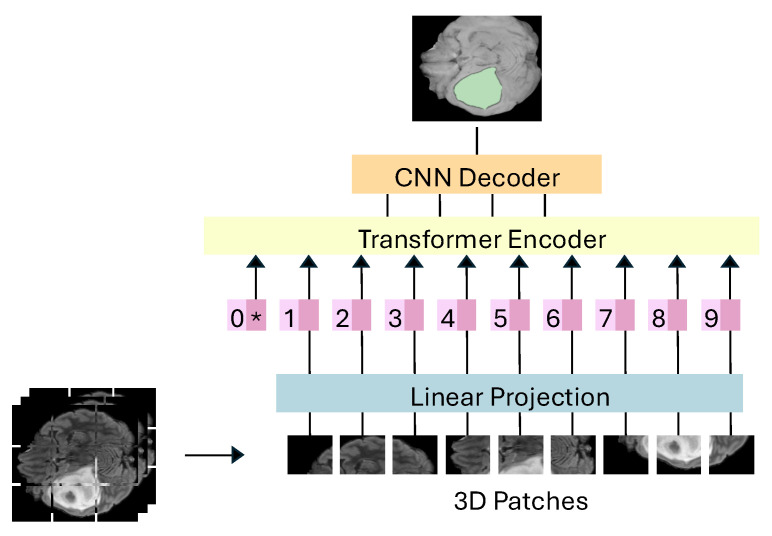
Hybrid CNN-Transformer Architecture. Merges CNN-based feature extraction with Transformer-based global contextual understanding.

Hybrid CNN-Transformer architectures are designed to integrate the spatial precision of CNNs with the long-range context capture of Transformers (see Figure 4). This integration aims to enhance segmentation accuracy by leveraging both local and global features, addressing the complex demands of tumor segmentation [66,67].

The fusion of CNN and Transformer technologies was pioneered by TransBTS [68]. These models enhance contextual and spatial relationships, despite facing high computational demands [69,70]. In response, more efficient architectures have recently been developed [71,72] to better handle multimodal data and missing modalities.

UNETR [73] represents a shift towards fully integrating Transformer capabilities for volumetric data and outperforming many state-of-the-art methods in most medical segmentation tasks. However, it usually struggles with fine details. In contrast, CFNet [74] focuses on multimodal brain tumor segmentation using hierarchical feature fusion from coarse to fine, enhancing segmentation through cross-modality attention, multi-scale context perception, and multimodal feature fusion. Moreover, Net [75] and UNetFormer [76] also try to mitigate the problem by simplifying training and enhancing accuracy but typically can lead to overfitting on the training set. To mitigate this, approaches like SegTransVAE [77] and TransBTSV2 [78] have focused on feature capturing and reducing overfitting.

With the ongoing need for generalization, federated learning and ensemble methods have been explored [79,80], although they encounter practical challenges related to domain adaptation in clinical settings. Alternatively, BiTr-Unet [81], Swin UNETR [60,82] optimize CNN and Transformer integration for medical applications, trying to minimize the existing domain gaps of the training and testing sets. These advancements are further extended by [83,84], refining architectures and employing topology-aware losses to enhance segmentation coherence.

Emerging models explore novel encoding and fusion strategies to balance local and global information [85,86,87,88,89,90], improving segmentation of irregularly shaped tumors through more advanced attention modules [91,92] or recalibration of features [93,94,95].

TransXAI [96] introduces a hybrid approach combining Vision Transformers (ViT) [97,98,99,100] and CNNs with explainable AI techniques to create interpretable heat maps, aiding medical professionals in understanding model decisions without compromising accuracy. In a more integrated application, SGS-Net [101] combines glioma segmentation with survival risk prediction, training both a segmentation decoder and a Cox model for survival analysis concurrently.

Further advancements are seen in models like 3D U-TFA [102], which incorporates Transformers and Feature Enhancement Attention within a 3D U-Net architecture to detect GB with vague boundaries more accurately. On the other hand, Arouse-Net [103] employs dilated convolutions and a focused attention mechanism on tumor edges, achieving faster processing times and improved metrics compared with most of the state-of-the-art models.

Finally, SAM [104,105,106,107,108] revolutionizes the standards towards prompt-driven interactive foundational models. Moreover, many methods have focused their research on efficient fine-tuning and optimization of SAM variants [109,110,111]. However, SAM is built to process 2D images, hence posing a challenge for volumetric medical data. To address this, Ref. [7] adapts the SAM2 [112] model for video semantic segmentation to be applied in the clinical domain.

These advancements illustrate a trend toward more generalizable, efficient, and interpretable segmentation solutions, addressing the complexities of brain tumor imaging with sophisticated architectural innovations.

### 3.4. Clinical Deployment

Despite the significant advancements in DL models for glioma segmentation, their deployment in real-world clinical environments remains limited due to a set of persistent challenges [113]. Although most models report strong performance on public datasets, these curated collections usually do not reflect the variability, artifacts, and limitations of routine hospital imaging [114]. As a result, the transition from research prototypes to clinically viable systems demands more than high segmentation accuracy; it requires robustness, efficiency, and practical integration capabilities.

One of the primary barriers to clinical deployment is the heterogeneity of MRI data across institutions [115]: differences in scanner hardware, acquisition protocols, image quality, and sequence availability can significantly degrade model generalization. Several studies highlighted this issue by comparing DL performance on retrospective clinical data against standardized datasets [116], revealing a substantial drop in segmentation accuracy when models trained on curated data were exposed to real-world inputs, reinforcing the importance of training on heterogeneous, multi-institutional datasets to ensure domain robustness.

Postoperative segmentation poses an additional challenge due to anatomical distortions, blood artifacts, resection cavities, and inconsistent sequences [117]. Algorithms initially designed for preoperative use tend to fail in these altered scenarios. Addressing this, some studies, such as RH-GlioSeg-nnU-Net [118], develop models specifically trained on postoperative data. On the other hand, Ref. [119] incorporates uncertainty estimation and non-tumorous tissue segmentation to improve reliability and clinical utility. Moreover, it provides an interactive tool enabling clinicians to visualize uncertainty maps and correct predictions in a short time, highlighting real-world usability.

Label quality has also proven critical, as ground truth manual segmentation is highly susceptible to inter-observer variability [115]. Due to this, uncertainty-aware loss functions and inter-observer validation have been proposed to mitigate label noise and improve reproducibility in training.

From a practical standpoint, computational constraints must be considered for deployment in routine workflows. Several models address this by employing lightweight architectures or hybrid pipelines that operate efficiently on standard hardware [111], avoiding the need for high-end GPUs. For example, Ref. [120] proposes a cascaded CNN with auto-encoder regularization that achieves full-case processing in under 10 min, including data routing, preprocessing, and segmentation. Other models eliminate the need for MRI-CT registration or minimize input requirements by demonstrating high segmentation accuracy using only T1c images [121].

In conclusion, the successful clinical deployment of DL-based glioma segmentation methods depends not only on segmentation accuracy but also on their robustness to variable data, resilience in postoperative settings, efficient resource use, and seamless integration into clinical systems. Importantly, low computational cost and reduced model complexity (i.e., limiting the number of trainable parameters) enable smoother integration into hospital environments with constrained resources, while also facilitating continuous refinement of the models over time as new clinical data becomes available.

## 4. Performance Analysis

In this section, we analyze the performance of more than 80 Deep Learning methods for glioma segmentation, focusing on the main publicly available databases for this task from the BraTS challenge collections. The analysis begins with a broad quantitative comparison of the different methods in terms of Dice Score [122], Hausdorff Distance [123], and the number of trainable parameters. Building on this review, we then identify the highest-performing models (those achieving a mean Dice of 90.00% or higher) for a focused qualitative evaluation. This subsequent analysis assesses their strengths, limitations, and overall suitability against several criteria for real-world clinical applicability. The ultimate goal is to synthesize the current state-of-the-art and inform a discussion on the most promising candidates for deployment in clinical environments based on specific operational requirements.

### 4.1. Glioma Databases

**Figure 5:**
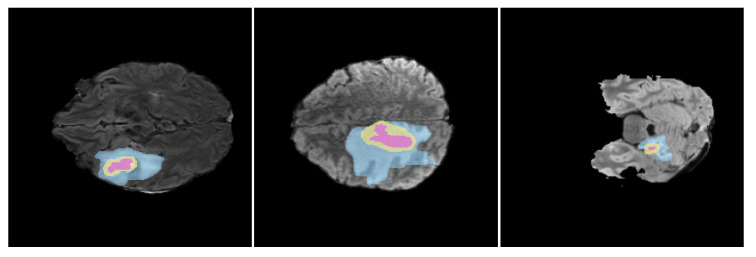
Glioma Structure. This figure displays segmented gliomas on MRI scans, where color coding is used to highlight different tumor regions: blue is the edema (ED), green denotes enhancing tumor (ET), and pink is necrotic cells (NCR). The joint of the ET and NCR areas represents the tumor core region (TC), while the union of the three segmented zones creates the whole tumor (WT).

In the field of medical imaging, databases such as The Cancer Imaging Archive (TCIA) [124] and the Human Protein Atlas [125] provide invaluable data across various types of cancers, including brain tumors. For glioma MRI imaging specifically, BraTS (Brain Tumor Segmentation Challenge) [4], The Cancer Genome Atlas (TCGA) [126], and the Federated Tumor Segmentation (FeTS) [127] are particularly notable. BraTS stands out as the most extensive and frequently used in the development of Deep Learning algorithms for brain tumor image analysis, offering a rich and complete dataset of annotated brain MRI glioma scans and differentiating three tumor regions: the Whole Tumor (WT), which encompasses the full extension of the glioma; the Enhancing Tumor (ET), which captures active cells of the tumor that are infiltrating surrounding tissue; and the Tumor Core (TC), which combines ET with necrotic cells, typically placed in the center of the glioma (see Figure 5).

Over the years, as shown in Table 1, the scale of the BraTS datasets has significantly increased in terms of the number of training, testing, and validation cases from 2012 to 2024, which allows for a broader evaluation of algorithm performance [23,128,129,130].

This paper will utilize the BraTS benchmark to compare the different state-of-the-art methods, as it represents the most standardized dataset for glioma MRI imaging.

### 4.2. Segmentation Metrics

In glioma segmentation research, the literature employs metrics such as sensitivity, specificity [131], precision, F1 score, and the Jaccard index [132] to evaluate algorithm performance. However, the Dice Score [122] and the Hausdorff Distance with a confidence score of 95% (HD95) [123] are the most widely accepted metrics to assess segmentation effectiveness, particularly in the BraTS challenge [133,134]. The Dice Score (described in Equation (1)) measures the overlap between the predicted segmentation and the actual tumor, reflecting accuracy in tumor volume delineation, while HD95 (described in Equation (2)) evaluates the maximum distance at the worst-case boundary points between the predicted and actual segments.

(1) $$\mathrm{Dice}\left(A,B\right)=\frac{2|A\cap B|}{\left|A|+|B\right|}$$

where

- *A* is the set of voxels in the ground truth segmentation.
- *B* is the set of voxels in the predicted segmentation.
-
|A| and |B| are the number of voxels in each set.
-
|A∩B| is the number of voxels that both segmentations share (overlap).

(2) $${\mathrm{HD}}_{95}\left(A,B\right)=\max\left\{{\mathrm{percentile}}_{95}\left(\min_{b\in B}d\left(a,b\right)\right),{\mathrm{percentile}}_{95}\left(\min_{a\in A}d\left(b,a\right)\right)\right\}$$

where

- *A* and *B* are the sets of boundary points from the ground truth and predicted segmentations, respectively.
-
d(a,b) is the Euclidean distance between points a∈A and b∈B .
-
$\min_{b\in B}d\left(a,b\right)$ is the minimum distance from a point *a* to any point in *B*.
-
${\mathrm{percentile}}_{95}$ denotes the 95th percentile of all such minimum distances.

In this paper, we use the Dice Score and the HD95 in order to compare different Deep Learning algorithms for glioma segmentation. Additionally, we study the number of trainable parameters for each method, considering the balance between model complexity and performance to determine their suitability for clinical applications. This approach helps identify models that are not only effective but also practical for deployment in medical settings.

### 4.3. Quantitative Review of Existing Methods

The segmentation of gliomas using Deep Learning methods has progressed significantly, driven by advancements in several architectures evaluated in the BraTS challenges.

CNN approaches, as presented in Table 2, typically achieve Dice Scores that mostly exceed 80%, with notable performers [44,135] reaching up to 95% in certain sub-regions, indicating high accuracy in tumor volume segmentation. However, the majority of these CNN-based approaches also exhibit high variation in HD95 distances, suggesting differences in their ability to precisely delineate tumor boundaries. Moreover, the underlying models vary greatly in their number of trainable parameters, ranging from as few as 6.92 M to over 111 M, suggesting the difficulty in trade-off between model performance and computational load.

Pure Transformer methods, as seen in Table 3, tend to maintain competitive Dice Scores but yet achieve lower performance compared with CNNs. Moreover, they often require a significantly higher number of tunable parameters, as seen in models like the HRSTNet-V4 [63] (266.33 M). This suggests that, while Pure Transformer approaches are capable of capturing complex spatial relationships, they do so at the expense of increased computational demand.

As depicted in Table 4, Hybrid CNN-Transformer algorithms showcase high Dice Scores and manage to keep HD95 distances low with comparable results to those achieved by CNNs while containing parameter counts to more manageable levels in many cases. For instance, GBT-SAM [111] achieves a Dice Score of 93.54% for the WT region with just 6.40 M trainable parameters, illustrating the effectiveness of integrating both architectures.

In conclusion, CNN-based approaches remain a strong baseline for their parameter efficiency and robust performance. Those based on Pure Transformers, while powerful, face challenges in deployment due to their high parameter counts. Alternatively, hybrid architectures offer a promising efficiency versus effectiveness compromise, suggesting that future developments could benefit from further exploration of this integrated approach.

### 4.4. Candidates for Real Deployment

While a vast number of deep learning models have been proposed for glioma segmentation, achieving high accuracy is only the first step toward clinical integration, while practical deployment requires models to be not only precise but also robust, efficient, and comprehensive. To this end, we provide a curated selection of models that demonstrate outstanding segmentation capability, defined as achieving a mean Dice Score of 90.00% or higher across all evaluated tumor regions. The goal is to analyze the strengths and limitations of these top-performing models to determine their viability as clinical support tools.

Table 5 presents a detailed comparison of these candidates. Beyond their architectural description, we evaluate them against four crucial conditions that directly impact their practical utility for assisting radiologists:

- (C1) Robustness to Data Variability: A method is considered robust if it has been validated on at least two different datasets or incorporates specific domain generalization techniques to ensure stable performance across varied clinical data.
- (C2) Postoperative/Low-Quality Data Handling: This condition marks methods designed or proven to handle challenging data, such as images with post-surgical changes or those from low-field or older imaging scanners, which can be encountered in clinical workflows.
- (C3) Computational Efficiency: A model is deemed efficient for this comparison if it has fewer than 50 million trainable parameters. Where available, we also include specific metrics like GFLOPs and inference latency to provide a clearer picture of the required computational resources.
- (C4) All Tumor Regions Delineated: This indicates whether the model provides segmentation for all three key sub-regions (WT, TC, and ET), as comprehensive delineation is essential for accurate diagnosis and treatment planning.

This structured evaluation aims to bridge the gap between theoretical performance and clinical applicability, highlighting models that best balance accuracy with the practical demands of a hospital environment.

**Table 5:** Comparison of glioma segmentation methods for clinical deployment readiness. The conditions (C1, C2, C3, C4) refer to (C1) Robustness to Data Variability, (C2) Postoperative/Low-Quality Data Handling, (C3) Computational Efficiency, and (C4) all tumor regions are delineated. The symbol “✓” indicates that the method addresses the condition.

Method	Description + Strengths	Limitations	C1	C2	C3	C4
Dense Unet+ [44]	CNN-based. Effective 4-modality MRI fusion; Weighted connections reduce semantic gaps; ROI-focused training for efficiency; Lower computational complexity for inference ( 1.16 s in convolutional layers).	Longer training time than baseline UNet; ResBlock coefficients require further optimization.	✓		✓	✓
Kuntiyellannagari et al. [135]	CNN-based. Advanced noise reduction via hybrid filter; Ensemble of three models for improved accuracy; Novel optimization algorithm (MAVOA) refines results.	Limited generalizability and interpretability; High computational demand from its hybrid filter.		✓		✓
Enhanced Unet [142]	CNN-based. Good generalization across multiple datasets; Optimized and simple architecture without complex additions; High computational efficiency ( 1.5 s per image).	Exclusive reliance on the FLAIR modality; Needs validation on a wider range of glioma grades; Only provides results for WT tumor region.			✓	
CBAM-TransUNet [146]	Hybrid CNN-Transformer. Combines U-Net, Swin Transformer, and CBAM; Attention module at the bottleneck to focus on key features; Includes a thorough robustness and ablation analysis.	Aggressive data cropping; Use of a fixed, non-optimized weight in the loss function; Limited reproducibility.				✓
Futrega et al. [83]	Hybrid CNN-Transformer. Extensive ablation study; Includes a highly optimized post-processing strategy.	Highly specialized for BraTS21; Only provides results for WT tumor region.			✓	
Unet Former [76]	Hybrid CNN-Transformer. Self-supervised pretraining for 3D medical images; Fully reproducible; Flexible architecture offering an accuracy and efficiency ( 149.50 GFLOPs) trade-off.	pretraining requires massive datasets and high-end hardware; pretraining effectiveness only tested on CT scans.	✓			✓
GBT-SAM [111]	Hybrid CNN-Transformer. Generalization across four tumor domains; Multimodal integration of full mp-MRI; Modeling 3D inter-slice correlation; Parameter-efficient method (6.4 M trainable parameters).	Dependent on bounding box prompts; Architecture specialized for four-modality MRI; Only provides results for WT tumor region.	✓	✓	✓	
CFNet [74]	Hybrid CNN-Transformer. Modules for coarse-to-fine multimodal feature fusion; Rigorous ablation studies; Fully reproducible; High computational efficiency (35.47 GFLOPs).	Fails to address the impact of MRI artifacts; Operates on 2D slices, losing direct 3D volumetric context.				✓
MAT [148]	Hybrid CNN-Transformer. Three-dimensional architecture using axial attention and self-distillation training that improves performance on small datasets; Smoother segmentation boundaries; Fully reproducible; Lightweight approach (11.7 M parameters).	Non-isotropic image resizing during preprocessing can distort anatomical geometry; Limited ablation studies.			✓	✓
Arouse-Net [103]	Hybrid CNN-Transformer. Attention mechanism specifically enhances tumor edges; Effective use of dilated convolutions to expand the receptive field of the model; High computational efficiency (1 s inference time).	Insufficient experimental validation; Limited generalizability; Lacks reproducibility.			✓	✓

### 4.5. Discussion

**Figure 6:**
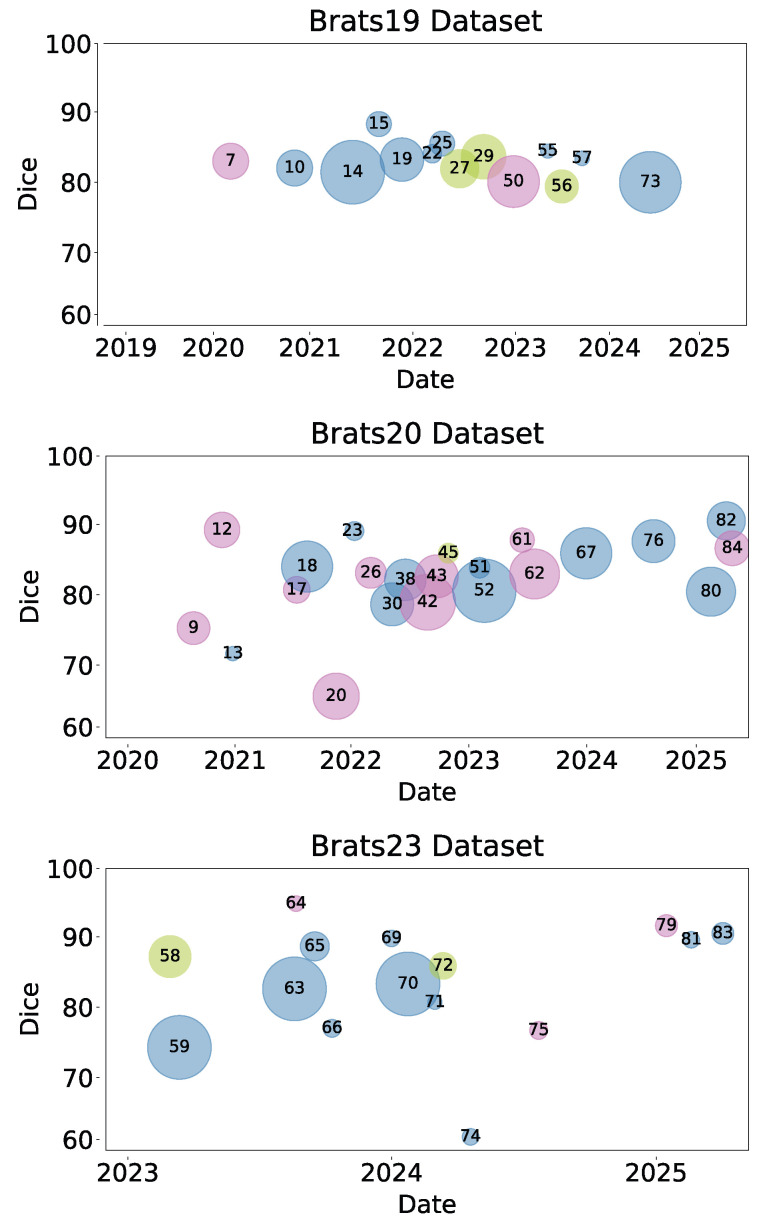
Dice Scores Across BraTS Datasets by Year. This figure shows Dice Scores for glioma segmentation within BraTS19, BraTS20, and BraTS23 datasets. Bubble size indicates the number of model trainable parameters, with color coding distinguishing between CNN (pink), Pure Transformer (green), and Hybrid CNN-Transformer (blue) architectures. This visualization highlights performance trends and model diversity over the years.

The analysis of Deep Learning methods for glioma segmentation across the three most used BraTS datasets is presented in Figure 6. This visualization chronologically maps different algorithms by their Dice Scores, with bubble size representing the number of trainable parameters and color indicating the architectural family. This figure, in conjunction with the detailed results in Table 2, Table 3 and Table 4, suggests significant trends in the evolution of CNN, Pure Transformer, and Hybrid models.

As observed, Hybrid architectures (blue bubbles) have become dominant in recent years, frequently achieving the highest Dice Scores. Notably, many of these top-performing models manage their computational complexity effectively, often using fewer parameters than older or less efficient models. This suggests a successful synergy, where the convolutional layers’ feature extraction capabilities are effectively enhanced by the transformers’ ability to model long-range dependencies, leading to improved segmentation accuracy. Conversely, CNN models (pink bubbles) demonstrate consistent and reliable performance, often with remarkable parameter efficiency. This makes them strong, practical candidates for clinical environments where computational resources may be limited. Pure Transformers (green bubbles), while powerful, show that higher parameter counts do not inherently guarantee superior Dice Scores, indicating that architectural optimization remains a key challenge for this model family.

While these performance metrics are crucial, the transition to a real-world clinical setting demands an analysis beyond Dice Scores. For this purpose, we evaluated the top-performing models (mean Dice ≥90 %) against four criteria for clinical readiness, as detailed in Table 5. A critical finding from this analysis is that no single method perfectly satisfies all conditions, hence revealing the gap that still exists between research benchmarks and clinical applicability. Based on this analysis, the choice of the optimal model is highly dependent on the specific clinical use case.

For workflows where the primary objective is a rapid, interactive segmentation of the whole tumor (WT), whether to guide surgery or for a quick initial assessment, GBT-SAM [111] emerges as a particularly compelling candidate. Its high parameter efficiency (C3), proven robustness across different data domains (C1), and ability to handle low-quality data (C2) make it highly adaptable. Its prompt-based interactive nature could also empower radiologists by keeping them directly involved in the segmentation process. However, this method does not provide delineation of different regions inside the tumor area.

Consequently, when the clinical need is a fully automated, comprehensive delineation of all tumor sub-regions (WT, TC, and ET) for detailed radiotherapy planning or treatment response assessment, Dense Unet+ [44] presents a more robust and suitable solution. It uniquely satisfies the crucial conditions of robustness to data variability (C1), computational efficiency (C3), and complete tumor segmentation (C4).

Ultimately, the selection of a tool must align with the specific needs, workflow, and available hardware of the implementing medical center. Every method analyzed in Table 5 has its own set of strengths and limitations. Therefore, significant research is still required.

## 5. Conclusions

Key advancements and current limitations in glioma segmentation are summarized in this section. Moreover, it includes a discussion on future research directions in this area.

### 5.1. Recent Developments and Current Limitations

In recent years, automatic glioma segmentation has experienced rapid progress through the development of pure Transformer architectures and hybrid CNN-Transformer models.

CNN-based models, such as 3D U-Net and V-Net, have notably advanced the handling of volumetric MRI data and delivered solid performance across multiple tumor subregions [39,40]. More adaptive designs like nnU-Net [45] have further enhanced versatility by introducing automatic architecture and preprocessing configurations tailored to specific datasets. However, performance variability observed across datasets such as BraTS17 through BraTS20 suggests that CNNs remain sensitive to dataset-specific distributions, raising concerns about their generalizability to diverse clinical scenarios. Notably, Dense Unet+ [44] achieved some of the best results overall, with a Dice score of 95.80 for the whole tumor region, 95.50 for the tumor core, and 93.70 for the enhancing tumor, outperforming many more recent architectures.

Pure Transformer-based models have introduced a new paradigm in medical image segmentation by effectively capturing long-range dependencies. Methods like VT-UNet [62] demonstrate this potential by achieving high Dice scores and low HD95 values in tumor core segmentation. Still, such improvements often come at the cost of large model sizes and increased computational demand.

Hybrid CNN-Transformer architectures like SwinBTS [2] and 3D U-TFA [102] have demonstrated state-of-the-art segmentation scores, with Dice(WT) exceeding 95 and competitive HD95 values. GBT-SAM [111], for example, achieved a Dice score of 93.54 (WT), alongside a surprisingly low parameter count of 6.4 million, reflecting the potential efficiency of hybrid designs, though such low complexity is not representative of most models in this category.

However, despite these impressive results on curated research datasets, our analysis reveals a critical gap between the high accuracy reported in research benchmarks and true medical applicability. The deployment of these models in real-world environments remains limited due to a persistent set of challenges [113] that include the poor generalization of models to heterogeneous clinical data, the inability to perform postoperative segmentation, and the demanding computational resources required by many state-of-the-art architectures [115]. Therefore, significant future research is needed to address these practical barriers, focusing on enhancing model robustness and efficiency to ensure the successful translation of these powerful tools into routine clinical practice.

### 5.2. Future Directions

Recent developments in glioma segmentation research highlight several key directions that are likely to shape future work. These directions reflect not only the evolution of model architectures and performance but also a growing emphasis on building systems that are practical, robust, and ready for real-edge deployment. Drawing on recent empirical patterns, three main research challenges emerge:

- Enhancing generalization across domains and datasets.
- Minimizing computational cost without compromising accuracy.
- Refining hybrid architectures for clinical integration.

First, generalization remains a considerable challenge. Deep learning models often suffer from “domain shift”, where a model trained on data from one hospital performs poorly when tested on data from another due to variations in scanners, acquisition protocols, and patient populations. This gap between performance on curated research data and real-world clinical data is a major barrier to adoption. Future research must prioritize methods that ensure models are robust to these variations. Two key strategies are emerging to address this:

- Domain Generalization techniques aim to train a single, robust model on data from multiple sources that can generalize well to completely unseen hospitals without needing retraining. This involves methods like advanced data augmentation, feature alignment, and disentanglement to learn domain-invariant features.
- Federated Learning offers a paradigm-shifting solution by training a shared global model across multiple institutions without ever centralizing or sharing sensitive patient data. Each institution trains the model locally, and only the model updates (weights or gradients) are sent to a central server for aggregation. This approach not only preserves data privacy but also naturally exposes the model to a diverse range of data, which has been shown to significantly boost the performance and robustness of brain tumor segmentation models. Validating models trained with these methods across multiple independent institutions (cross-institutional validation) is becoming the gold standard for proving their real-world clinical readiness.

Second, improving computational efficiency remains essential for real-world deployment. Although historically the best-performing models have relied on large parameter counts, recent trends suggest that competitive segmentation accuracy can be achieved with more compact designs. This opens the door for research into model compression, lightweight attention mechanisms, and neural architecture search aimed at producing architectures that are both high-performing and suitable for resource-constrained hardware.

Finally, as this review has shown, hybrid CNN-Transformer architectures have become increasingly prominent and now dominate the upper range of segmentation performance. Their flexibility positions them as strong candidates for clinical translation. Future directions will likely focus on modular, adaptive variants of these hybrids, possibly enhancing model transparency through interpretability and uncertainty estimation frameworks to build greater trust with clinicians.

## Figures and Tables

**Table 1:** Evolution of the BraTS Challenge Datasets. This table summarizes the annual distribution of training, validation, and testing cases within the BraTS benchmark from 2012 to 2024, illustrating the expansion and evolution of the dataset over time.

Year	Total Cases	Train	Validation	Test
2012	50	35	N/A	15
2013	60	35	N/A	25
2014	238	200	N/A	38
2015	253	200	N/A	53
2016	391	200	N/A	191
2017	477	285	46	146
2018	542	285	66	191
2019	626	335	125	166
2020	660	369	125	166
2021	2040	1251	219	570
2022	2000	1251	219	530
2023	2040	1251	219	570
2024	2200	1540	220	440

**Table 2:** CNN-based Approaches. This table compares CNN-based algorithms for glioma segmentation, grouped chronologically by dataset. It reports the Dice Scores for Whole Tumor (WT), Enhancing Tumor (ET), and Tumor Core (TC); the 95th percentile Hausdorff Distance (HD95); and the number of trainable parameters (in millions). The best-performing method per metric and dataset group (when more than one method is available) is highlighted in bold. Note: An asterisk (*) indicates that the number of parameters was not explicitly reported by the authors but was estimated based on the model architecture. ↑↓: Lower or upper values are better for each metric.

Id	Algorithm	Dice(WT)↑	Dice(ET)↑	Dice(TC)↑	HD95↓	#Params(M)↓	Dataset
3	Wang et al. [42]	90.50	78.59	83.78	16.50	20.00 *	Brats17
4	Att. Unet [55]	87.24	74.51	76.85	7.61	34.90	Brats18
6	Myronenko [53]	**91.00**	**82.33**	**86.68**	5.10	7.70 *	Brats18
8	DenseMultiOCM [136]	86.00	73.20	73.33	8.75	35.00 *	Brats18
39	DPAFNet [137]	90.50	79.50	83.90	**5.05**	28.00 *	Brats18
41	MSFR-Net [138]	90.90	80.70	85.80	4.82	37.85	Brats18
1	3D U-Net [39]	85.36	72.21	71.05	12.94	19.00	Brats19
2	V-Net [40]	85.18	72.43	73.46	8.92	37.70	Brats19
7	TuNet [41]	**90.34**	**78.42**	81.12	**4.77**	30.50 *	Brats19
50	Tong et al. [50]	88.50	75.10	77.60		61.84	Brats19
86	AR-CA [120]	83.00	72.00	**84.00**		15.00 *	Brats19
5	nnUnet [45]	90.70	81.40	84.80	5.95	41.20	Brats20
9	RFNet [46]	86.98	61.47	78.23		35.00 *	Brats20
12	Modality-pairing [48]	92.40	86.30	89.80	**4.92**	42.00 *	Brats20
17	MM-BiFPN [51]	83.58	77.95	81.47		21.41	Brats20
20	MAF-Net [47]	88.00	41.80	67.90		75.00 *	Brats20
26	Dres-Unet [43]	86.60	80.04	83.57		30.47	Brats20
42	MM-Unet [57]	85.00	76.20	76.50	8.47	111.40	Brats20
43	Liu et al. [49]	89.50	77.45	81.78	6.40	64.00 *	Brats20
61	Sahoo et al. [54]	90.36		85.75		17.00 *	Brats20
62	GAM-Net [52]	89.91	75.80	84.02	5.30	88.00 *	Brats20
78	Kuntiyellannagari et al. [135]	**97.00**	**91.00**	**96.00**		147.00 *	Brats20
84	Rastogi et al. [139]	87.56	86.46	86.66		40.00 *	Brats20
87	GBManalizer [140]	89.33	80.69	83.72	6.84	**0.004**	Brats20
88	RH-GlioSeg-nnU-Net [118]	88.00	78.00	72.00		41.20 *	Brats20
35	MAAB [56]	89.07	78.02	80.73	19.59	24.00 *	Brats21
64	Dense Unet+ [44]	**95.80**	**93.70**	**95.50**		**6.92**	Brats23
75	Beser-Robles et al. [141]	71.00	81.00	79.00		19.00	Brats23
79	Enhanced Unet [142]	91.87			4.12	46.00 *	Brats23

**Table 3:** Pure Transformer Approaches. This table compares Transformer-based algorithms for glioma segmentation, grouped chronologically by dataset. It reports Dice Scores for Whole Tumor (WT), Enhancing Tumor (ET), and Tumor Core (TC); the 95th percentile Hausdorff Distance (HD95); and the number of trainable parameters (in millions). The best-performing method per metric and dataset group (only when more than one method is available) is highlighted in bold. Note: An asterisk (*) indicates that the number of parameters was not explicitly reported by the authors but was estimated based on the model architecture. ↑↓: Lower or upper values are better for each metric.

Id	Algorithm	Dice(WT)↑	Dice(ET)↑	Dice(TC)↑	HD95↓	#Params(M)↓	Dataset
28	Pinaya et al. [143]	61.70				48.91 *	Brats18
77	MMCFormer [65]	**85.00**	**64.70**	**79.20**		**8.57**	Brats18
27	BTSwin-Unet [59]	**90.28**	78.38	81.73	**5.18**	35.60	Brats19
29	EMSViT [61]	**90.28**	**79.24**	**82.23**	5.49	47.50 *	Brats19
56	Swin-Unet [60]	86.89	75.62	76.63	7.89	**27.10**	Brats19
45	NestedFormer [58]	92.20	80.00	86.40	5.05	10.48	Brats20
44	VT-Unet [62]	92.24	86.31	89.53	3.51	87.00	Brats21
58	HRSTNet-4 [63]	**91.90**	**82.92**	**87.62**	**8.94**	266.33	Brats23
72	CR-Swin2-VT [64]	91.38	81.71	85.40	9.97	90.00 *	Brats23

**Table 4:** Hybrid CNN-Transformer Approaches. This table compares Hybrid CNN-Transformer algorithms for glioma segmentation, ordered chronologically by dataset. It reports Dice Scores for Whole Tumor (WT), Enhancing Tumor (ET), and Tumor Core (TC); the 95th percentile Hausdorff Distance (HD95); and the number of trainable parameters in millions. The best-performing method per metric and dataset group (only when more than one method is available) is highlighted in bold. Note: An asterisk (*) indicates that the number of parameters was not explicitly reported by the authors but was estimated based on the model architecture. ↑↓: Lower or upper values are better for each metric.

Id	Algorithm	Dice(WT)↑	Dice(ET)↑	Dice(TC)↑	HD95↓	#Params(M)↓	Dataset
40	ERTN [144]	83.20	73.59	77.93	5.13	95.00 *	Brats17
16	Zhou et al. [72]	82.90	59.10	74.90	7.10	37.00 *	Brats18
46	mmFormer [86]	**89.60**	85.80	77.60	7.85	106.00	Brats18
47	LGMSU-Net [91]	87.35				69.02	Brats18
53	Tongxue Zhou [89]	86.50	**87.00**	**79.40**	**3.60**	**36.00**	Brats18
10	TransBTS [68]	88.42	77.58	80.96	7.17	30.60	Brats19
14	SegTran [70]	89.50	74.00	81.70		93.10	Brats19
15	Fang et al. [71]	**92.67**	**83.54**	**89.47**	**1.95**	15.37	Brats19
19	Transition-Net [75]	91.25	74.85	84.46	14.15	44.00 *	Brats19
22	TransConver [66]	90.19	78.40	82.57	4.74	9.00	Brats19
25	TransBTSv2 [68]	90.42	80.24	84.87	4.87	15.30	Brats19
55	Gao et al. [94]	90.10	80.10	84.00	4.73	**5.90**	Brats19
57	GMetaNet [95]	90.20	78.40	82.50	4.84	6.10	Brats19
73	TransXAI [96]	88.20	74.50	78.20	6.19	87.00 *	Brats19
13	Medical Transformer [69]	87.33	58.82	69.69		**2.41**	Brats20
18	UneTR [73]	89.90	78.80	84.20	5.25	92.58	Brats20
23	TranSiam [67]	89.34			5.65	7.98	Brats20
30	Nalawade et al. [79]	87.40	72.10	77.30	27.09	64.00 *	Brats20
38	RMTF-Net [85]	81.80				59.00 *	Brats20
51	AST-Net [87]	90.40	77.80	84.20	14.23	10.50	Brats20
52	Huang et al. [88]	90.30	70.80	81.50	15.99	144.86	Brats20
67	TransDoubleU-Net [90]	92.87	79.16	86.51	10.77	93.00 *	Brats20
68	GSG U-net [145]	91.28	85.88	85.82	5.39	60.00 *	Brats20
76	SwinBTS [2]	**95.06**	85.36	83.30	10.03	64.00 *	Brats20
80	SGS-Net [101]	89.79	76.12	76.29		85.00 *	Brats20
82	CFNet [74]	91.60	**90.29**	**90.46**	**1.51**	49.18	Brats20
21	Unet Former [76]	**93.22**	**88.80**	92.10	8.49	58.96	Brats21
24	SegTransVAE [77]	90.52	85.48	**92.60**	4.10	44.70	Brats21
31	Shi et al. [80]	89.15	81.94	73.81	13.09	143.00 *	Brats21
32	BiTr-Unet [81]	90.97	81.87	84.34	13.01	43.50	Brats21
33	Dobko et al. [82]	86.98	84.96	92.56	10.05	94.00 *	Brats21
34	Futrega et al. [83]	91.63				43.00 *	Brats21
36	Swin UneTR [3]	92.60	85.80	88.50	5.21	61.98	Brats21
37	COTRNet [84]	89.34	77.60	80.21	17.24	46.51	Brats21
48	CBAM-TransUNet [146]	93.08	87.76	91.49	**4.01**	96.00	Brats21
49	3D PSwinBTS [92]	92.64	82.62	86.72	10.78	**20.40**	Brats21
54	AABTS-Net [93]	91.10	77.70	83.80	4.42	75.00 *	Brats21
60	Med-SA [109]	88.70			9.50	13.00	Brats22
59	SAM [104]	74.60			27.51	636.00	Brats23
63	SAM-Med2D [106]	82.90			16.20	636.00	Brats23
65	MFD-Net [147]	92.70	85.40	88.70	7.76	105.00 *	Brats23
66	SAMed [105]	77.30			19.07	18.81	Brats23
69	MAT [148]	93.21	85.05	**91.91**	**4.77**	11.70	Brats23
70	MedSAM [110]	83.60			14.90	636.00	Brats23
71	SAM-U [149]	81.00			17.26	**0.00**	Brats23
74	Diana-Albelda et al. [38]	61.90			32.00	12.00	Brats23
81	3D U-TFA [102]	**95.06**	85.36	83.30	10.03	11.31 *	Brats23
83	GBT-SAM [111]	93.54				6.40	Brats23
85	Arouse-Net [103]	93.50	**89.30**	89.50		45.00 *	Brats23
